# Biomechanical Applications of Finite Element Analysis in Orthodontics: A Scoping Review of Force Distribution, Tooth Movement, and Mechanical Performance

**DOI:** 10.3390/dj14030148

**Published:** 2026-03-06

**Authors:** Valenciana-Solís Jesús Antonio, Gaitán-Fonseca César, Flores Héctor, Zavala-Alonso Verónica, Bermúdez-Jiménez Carlos, Martínez-Torres Carlos, Pozos-Guillén Amaury

**Affiliations:** 1 Doctorado Institucional en Ingeniería y Ciencia de Materiales, Universidad Autónoma de San Luis Potosí, San Luis Potosí 78210, Mexico; a396520@alumnos.uaslp.mx (V.-S.J.A.); heflores@uaslp.mx (F.H.); 2 Unidad Académica de Odontología, Universidad Autónoma de Zacatecas “Francisco García Salinas”, Zacatecas 98600, Mexico; cgaitan@uaz.edu.mx (G.-F.C.); carlosber8@uaz.edu.mx (B.-J.C.); 3 Facultad de Estomatología, Universidad Autónoma de San Luis Potosí, San Luis Potosí 78290, Mexico; nveroza@fest.uaslp.mx; 4 Independent Researcher, León 37358, Mexico; ortokarlos@gmail.com

**Keywords:** finite element analysis, orthodontics, orthodontics biomechanics, digital orthodontics, scoping review

## Abstract

**Background/Objectives:** Clinical and scientific professionalization in orthodontics requires a comprehensive understanding of the biomechanical principles governing force generation and distribution produced by orthodontic appliances, beyond purely esthetic considerations. In this context, finite element analysis (FEA) has emerged as a fundamental computational tool for the detailed evaluation of the biomechanical behavior of the dentoalveolar system. The aim of this study was to map and synthesize the available scientific evidence on the application of FEA in the assessment of force distribution, tooth movement, and the mechanical response of periodontal tissues during orthodontic treatment. **Methods:** Original studies published between 2020 and 2025 that relied exclusively on computational simulations using FEA were included. Eligible studies addressed orthodontic biomechanics, including tooth movement, appliance–tooth–periodontium interactions, or the mechanical evaluation of orthodontic attachments. Clinical studies, narrative reviews, and articles without finite element modeling were excluded. A systematic literature search was conducted in the PubMed and ScienceDirect databases to answer the following question: Which FEA methodologies have been used to evaluate the biomechanical behavior of orthodontic appliances? **Results:** Data were categorized according to key biomechanical variables. The findings indicate an increasing use of FEA as a supportive tool in orthodontic research. However, significant limitations were identified, including lack of methodological standardization, limited model validation, and insufficient correlation between computational outcomes and clinical evidence. **Conclusions:** Currently, FEA in orthodontics is used predominantly for descriptive purposes, particularly for visualizing stress and strain distributions. Greater standardization and validation are required to enhance its translational applicability in clinical relevance.

## 1. Introduction

Clear aligners (CAs) have gained increasing acceptance as an alternative to conventional fixed orthodontic appliances due to their esthetic appeal, patient comfort, and ease of use [1]. Nevertheless, the clinical and scientific professionalization of orthodontic practice requires moving beyond esthetic considerations toward a deeper understanding of the biomechanical behavior of orthodontic devices. In particular, it is essential to analyze how the force systems generated by these appliances are distributed and transmitted to dental and periodontal structures, as well as how material properties influence their performance and associated biological responses [2].

When mechanical loads are applied to a structure, internal stresses and deformations are generated; however, these variables cannot be directly measured, especially in complex biological systems. The stomatognathic system represents a highly intricate biomechanical environment, composed of teeth, periodontal ligament (PDL), alveolar bone, and surrounding tissues that interact dynamically under functional and therapeutic loads. Due to ethical, biological, and methodological limitations associated with in vivo experimentation, orthodontic biomechanics relies heavily on in vitro methodologies and computational modeling to predict tissue behavior and mechanical responses [3]. Orthodontic biomechanics encompasses the mechanical principles governing tooth movement across a wide range of appliances, including fixed brackets, clear aligners, skeletal anchorage systems, and hybrid mechanics. Orthodontic tooth movement results from controlled-force systems applied to teeth, inducing mechanical stimulation and subsequent biological remodeling within the PDL and alveolar bone [4]. Previous studies have extensively described these biomechanical and biological responses, emphasizing the critical role of force magnitude, direction, and point of application in achieving predictable, efficient and biologically safe tooth movement [3]. Orthodontic tooth movement is not purely mechanical but results from a complex biologically mediated process. Force application induces PDL deformation, vascular changes, and cellular signaling pathways that regulate bone resorption and apposition. The viscoelastic behavior of the PDL and the time-dependent nature of bone remodeling are critical determinants of clinical outcomes, yet these adaptive processes are often simplified or omitted in computational simulations [5].

In recent years, finite element analysis (FEA) has emerged as a powerful and widely adopted tool in orthodontic biomechanics research [6]. This computational approach allows the simulation of complex anatomical geometries and material properties, enabling detailed evaluation of force distribution, stress and strain patterns, tooth displacement, and the mechanical behavior of periodontal tissues. As a result, FEA has become instrumental in advancing the understanding of orthodontic mechanics and supporting the optimization of treatment strategies with greater precision and predictability [7,8]. While clear aligners represent an increasingly studied modality, finite element analysis (FEA) has been applied broadly to evaluate force distribution, periodontal stress response, and displacement behavior across diverse orthodontic treatment strategies.

The present study aims to conduct an exploratory review of the existing literature on the application of FEA in orthodontics, with particular emphasis on force distribution, tooth movement patterns, and the mechanical evaluation of different orthodontic systems. By synthesizing current evidence, this scoping review seeks to provide a comprehensive overview that supports the development of more efficient, biologically sound, and predictable approaches in contemporary orthodontic practice.

## 2. Methods

### 2.1. Design

A scoping review was selected as the methodological design for this study, as it is particularly suitable for mapping and synthesizing the existing literature across a broad range of key concepts related to a clinical topic of interest [9]. This type of approach is especially valuable when the accessible evidence is heterogeneous, methodologically diverse, or has not yet been comprehensively reviewed, thereby limiting the feasibility of conducting a more narrowly focused systematic review [10,11,12]; the Preferred Reporting Items for Systematic Reviews and Meta-analysis guidelines for Scoping Reviews (PRISMA-ScR) was also used [13].

A review protocol was developed *a priori* and registered in the Open Science Framework (OSF), where it is publicity accessible at https://doi.org/10.17605/OSF.IO/ED785, accessed on 24 January 2026. The protocol details the study objectives, eligibility criteria, information sources, search strategy, study selection process, and data charting methods.

The methodological framework for this scoping review followed established guidance and comprised five sequential stages: (i) formulation of a clearly defined research question; (ii) systematic searching for and identification of relevant studies; (iii) selection of eligible studies based on predefined inclusion and exclusion criteria; (iv) extraction and charting of relevant data; and (v) collation, synthesis, and structured reporting of the results.

### 2.2. Stage I: Formulation of the Research Question

The research question guiding this scoping review was formulated to capture the breadth of available computational evidence in orthodontic biomechanics: *Which finite element analysis methodologies have been used to evaluate force distribution, tooth movement, and the mechanical behavior of orthodontic appliances and periodontal tissues?* This intentionally broad question was designed to enable the identification of diverse modeling approaches, orthodontic systems, and biomechanical outcomes reported across the literature.

### 2.3. Stage II: Search Strategy and Identification of Relevant Studies

A comprehensive literature search was conducted in the PubMed and ScienceDirect electronic databases to identify relevant studies addressing the application of FEA in orthodontics. The search strategy combined controlled vocabulary and free-text terms related to computational modeling, orthodontic treatments, auxiliary devices, skeletal anchorage systems, hybrid force-delivery mechanics, orthodontic biomechanics, and biomechanical outcomes. The following search string was applied:

(Finite Element Analysis OR FEA) AND (Orthodontics OR Orthodontic Treatment) AND ((Clear aligner) OR (Fixed Orthodontic OR Brackets)) AND (Force Distribution OR Stress Analysis).

The review was restricted to studies published between 2020 and 2025 to specifically map contemporary applications of orthodontic finite element modeling. This period reflects recent advances in digital orthodontics, three-dimensional imaging integration, and aligner biomechanics, which have substantially influenced current research trends and clinical translation. Only articles published in English and appearing in peer-reviewed journals were considered eligible for further screening.

### 2.4. Stage III: Study Selection and Eligibility Criteria

Study selection was conducted in accordance with predefined inclusion and exclusion criteria. Eligible studies were required to employ FEA as the primary methodological approach, focus on orthodontic treatments involving clear aligners or fixed appliances, and present in silico simulations addressing biomechanical aspects such as force distribution, stress analysis, or tooth movement. Only studies that evaluated the mechanics of orthodontic treatment without auxiliary anchorage devices (tensoral anchorage devices, mini-implants or extraoral appliances) were included. This decision was made to reduce methodological heterogeneity and isolate the biomechanical effects generated solely by the primary orthodontic appliances. Studies that were exclusively clinical in nature, narrative or systematic reviews, letters to the editor, opinion articles or publications lacking a clear description of the finite element model were excluded. Additionally, studies not related to orthodontic applications or without a computational or biomechanical component were not considered.

**Figure 1:**
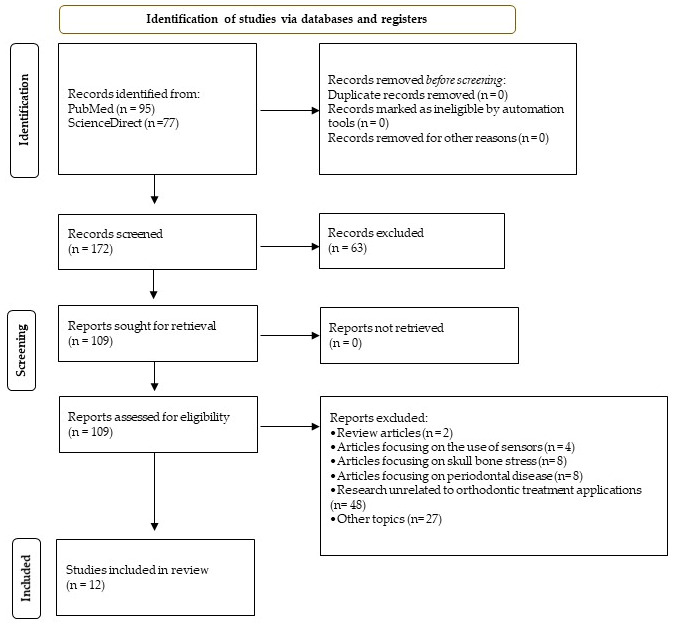
Literature search flowchart.

The screening and selection process was performed independently by three reviewers (AVS, CGF, and CBJ). Discrepancies in study eligibility were resolved through discussion and consensus. When consensus could not be achieved, four additional reviewers (APG, HFR, VZA, and CMT) independently evaluated the disputed articles to ensure an objective and balanced decision-making process. Although formal inter-reviewer agreement statistics were not calculated, this process was implemented to enhance methodological transparency and consistency. Following full-text assessment, a total of 12 studies met the eligibility criteria and were included in the final synthesis. The study selection process is summarized in Figure 1.

## 3. Results

### Stage IV and V: Data Extraction, Charting, and Synthesis of Results

The study selection process is summarized in the PRISMA-ScR flow diagram (Figure 1). Following the removal of duplicate records, the remaining articles were screened based on titles and abstracts. Studies considered potentially eligible underwent full-text assessment and those that did not meet the predefined inclusion criteria were excluded at this stage. Ultimately, 12 studies employing three-dimensional finite element models were included in the final analysis. Data extraction and charting were conducted systematically to capture key characteristics of each study, including the type of orthodontic appliance evaluated, modeling approach, material properties, loading conditions, and biomechanical outcomes assessed. Rather than focusing on isolated numerical magnitudes, the results highlight consistent biomechanical trends across studies, including patterns of incisor tipping during retraction, stress concentration zones within the PDL, and the influence of aligner design variables on movement predictability. The extracted information is summarized in Table 1.

All included studies focused on the biomechanical analysis of orthodontic treatment using FEA. The majority of them analyzed transparent aligners, whereas a smaller proportion examined conventional fixed appliances with brackets. Despite variations in appliance type and modeling assumptions, a considerable degree of methodological consistency was observed across the included studies. In all cases, FEA served as the primary analytical method for quantifying stress distribution, strain patterns, and force transmission within the dentoalveolar structures, enabling meaningful comparison among different orthodontic systems.

With respect to computational tools, most studies employed commercial finite element software packages including ANSYS, LS-DYNA, SpaceClaim, SOLIDWORKS, HyperMesh, ABAQUS and CREO. All models were constructed using three-dimensional geometries derived from cone-beam computed tomography (CBCT) images, providing anatomically realistic representations of teeth, PDL, and surrounding alveolar bone. This shared modeling framework facilitated the structured synthesis of the findings, allowing the identification of prevailing trends, methodological similarities, and existing gaps within the current literature.

The detailed characteristics and principal findings of each included study are described below.

Li et al. (2023) investigated force systems, stress distribution, and movement tendencies of mandibular teeth subjected to intrusion with clear aligners using three-dimensional finite element analysis. Owing to bilateral symmetry, the left mandibular dentition was selected as representative for the simulations. The center of resistance (CR) was defined at two-fifths of the distance from the alveolar crest and three-fifths from the root apex, in accordance with established biomechanical assumptions. In mandibular incisors with an incisor–mandibular plane angle (IMPA) of 90°, an incisal coronal moment was observed. With increasing IMPA, this moment decreased and shifted to a buccal moment, with an inflection point around 105°. von Mises stress was mainly concentrated at the lingual fossa and incisal edge, corresponding to primary aligner contact areas. As IMPA increased, compressive stress shifted from the lingual to the labial root surface, with similar patterns observed in the PDL. In mandibular canines, a constant distal crown moment was identified, accompanied by mesially directed and extrusive forces. Incisor IMPA variations had minimal influence on canine force systems. Stress was localized at the cusp tip, distolabial planes, and attachment regions, while tensile stress was concentrated in the cervical half of the root, particularly on the distal surface. Displacement analysis showed distal crown rotation with mesial apex displacement, lingual crown tipping, and a tendency toward extrusion. These patterns were attributed to clear-aligner deformation, inducing unintended labial and mesial root apex movement [14].

Kang et al. (2023) used three-dimensional FEA to evaluate force systems, displacement patterns, and PDL stress during en-masse retraction of maxillary incisors after premolar extraction. Three torque conditions were compared: small torque (ST, 100°), middle torque (MT, 110°), and high torque (HT, 120°). Across all groups, bilateral symmetry and consistent movement trends were observed. The incisors showed lingual tipping with extrusion, while the root apex displayed slight labial displacement. Incisal edge displacement was greatest in the ST group (9.92 × 10^−2^ mm) and decreased with increasing torque (MT: 8.30 × 10^−2^ mm; HT: 8.24 × 10^−2^ mm), indicating that lower torque produces greater incisal displacement. Canines exhibited minimal movement, characterized by lingual intrusion and mesial rotation. Posterior teeth showed mesial tipping with smaller and less uniform displacement. PDL stress was concentrated in the cervical region and root apex, decreasing progressively from incisors to molars. The authors concluded that clear-aligner en-masse retraction generates predictable displacement and stress patterns, influenced by incisal inclination and intrusion force magnitude [15].

Seo et al. (2021) used three-dimensional FEA to assess the biomechanical effects of clear aligners with two thicknesses (0.5 mm and 0.75 mm) during correction of lingual inclination and axial rotation of a maxillary central incisor. Both aligners produced comparable tooth movements, with slightly greater buccolingual inclination in the 0.5 mm model, while axial rotation outcomes were nearly identical between thicknesses. Stress analysis showed that the highest values were concentrated in the central incisor, whereas adjacent teeth exhibited minimal stress. During lingual-inclination correction, maximum PDL stress was mainly located in the apical region. Tensile stress predominated on lingual surfaces, while compressive stress was concentrated buccally. Overall, the thicker aligner generated moderately higher PDL stress levels (approximately 6%), reflecting increased rigidity and force transmission. These findings indicate that aligner thickness influences periodontal stress magnitude, although overall displacement patterns remain similar [16].

Jin et al. (2024) combined three-dimensional finite element modeling with experimental validation to examine force systems, displacement patterns, and periodontal stress distribution during clear-aligner therapy, focusing on aligner thickness and the incisor–mandibular plane angle (IMPA). During lingual-inclination correction, stress was primarily concentrated on lingual PDL surfaces, while compressive stresses were localized buccally. The thicker aligner (0.75 mm) generated moderately higher stress levels than the thinner model (0.5 mm), reflecting increased rigidity and force transmission. During axial rotation correction, stress concentration shifted toward the middle and cervical regions of the PDL, with only minimal differences in magnitude between aligner thicknesses. Center of rotation (COR) analysis revealed thickness-dependent positional changes, with both models showing lingual and apical COR displacement during inclination. In contrast, axial rotation produced divergent COR shifts between crown and root levels depending on aligner thickness. Overall, these findings highlight that aligner thickness influences stress distribution and rotational biomechanics, despite producing broadly comparable movement patterns [17].

Qiang et al. (2024) performed a three-dimensional FEA to compare two orthodontic treatment models designed to evaluate the lingual inclination of anterior teeth and the associated anchorage loss under different extraction patterns. Both models exhibited similar overall biomechanical trends, although the magnitude of the effects was greater in Model 1. In both configurations, the central incisors demonstrated the smallest degree of lingual inclination, whereas the greatest distal inclination was observed in the canines in Model 1 and in the first premolars in Model 2, indicating increased anchorage loss in these teeth. In the maxillary arch, the central incisor in Model 1 showed a lingual inclination of 0.1172°, which was approximately 2.7 times greater than that observed in Model 2 (0.0442°). PDL stress values were consistently higher in the maxilla than in the mandible. In Model 1, stress distribution was relatively uniform across the dental arch, with increased values in regions adjacent to the extraction spaces. The highest compressive stresses were identified in the distal cervical region and the mesial root apex of the canines, whereas peak tensile stresses were localized in the mesial cervical region and the distal apex. By contrast, Model 2 exhibited higher peak compression and tension stress values, predominantly concentrated in the first premolars. Total deformation was greatest in the incisor region and progressively decreased toward the molars, which exhibited the lowest deformation values, consistent with the applied loading conditions. von Mises stress was distributed relatively evenly between incisors and molars, with notable concentration in areas adjacent to the extraction sites. Overall, the findings underscore the influence of extraction pattern on force transmission, anchorage control, and stress distribution during clear-aligner-based orthodontic treatment [18].

Zhang et al. (2023) performed a three-dimensional finite element analysis to evaluate the biomechanical effects of maxillary expansion with clear aligners. Because bilateral teeth showed symmetrical displacement patterns, the right maxillary arch was used as representative, and the analysis focused on premolars and first molars, given the limited contribution of second molars to transverse expansion. During expansion, anterior teeth exhibited lingual tipping with incisor extrusion, whereas posterior expansion was mainly achieved through buccal tipping accompanied by slight distal inclination. Increasing step length intensified posterior tipping but reduced expansion efficiency, particularly from the first premolar to the first molar. Bodily movement of posterior teeth was only observed under specific pitch–torque combinations, requiring greater torque as pitch increased. At larger step lengths (0.3 mm), compensated torque had little effect, and expansion occurred predominantly through tipping rather than translation. von Mises stress was concentrated in the cervical root regions of the canine and first premolar, with PDL stress mainly localized cervically. Stress levels increased with higher torque angles and step length, although remaining within the same order of magnitude. Overall, the study highlights how aligner activation parameters strongly influence both movement patterns and periodontal stress distribution during maxillary expansion [19].

Zhu et al. (2024) evaluated five clear-aligner-based protocols for molar distalization using three-dimensional FEA. The five biomechanical protocols were defined as follows: Configuration A consisted of distalization of the second molar. Configuration B included distalization of the second molar combined with extrusion of the first molar. Configuration C comprised distalization of the second molar together with extrusion of the first molar and both the first and second premolar. Configuration D involved distalization of the second molar with extrusion of the first molar and the first and second premolars. Finally, Configuration E included distalization of the second molar, extrusion of the first molar and the first and second premolars, as well as expansion of the first molar and de first and second premolars. Across all configurations, mesial displacement of the root apices was observed, resulting in an uncontrolled distal–lingual tipping pattern of the molars. Analysis of tooth displacement along the x-axis revelated that Configuration A produced the greatest magnitude of movement (0.0937 mm), whereas Configuration E demonstrated the smallest displacement (0.0855 mm), indicating a measurable influence of distalization design on movement efficiency. The distribution of stress within the PDL varied according to tooth type and distalization configuration. In the central incisors, stress was predominantly concentrated in the cervical third of the lingual surface. For the lateral incisors, stress was mainly localized in the cervical region of the labial and distal surfaces. In the canines, stress distribution was configuration-dependent: in Configurations A and B, stress was concentrated in both the cervical third and the root apex; in Configurations C and D, stress was primarily confined to the cervical region; and in Configuration E, stress was distributed across the cervical, labial, and lingual regions. Overall, equivalent PDL stress in the anterior teeth was most frequently observed in the distal cervical and labial regions of the canine, with comparatively lower stress intensity at the root apex. Notably, in Configuration E, stress concentration was restricted to the cervical and labial regions of the canine. These findings demonstrated that different distalization protocols can substantially influence both the magnitude of tooth displacement and the distribution of biomechanical load within periodontal tissues during clear-aligner therapy [20].

Mao et al. (2023) investigated the biomechanical effects of two types of clear aligners, scanned aligners and ideal aligners, using three-dimensional morphologic assessment combined with finite element analysis. In both groups, mesiodistal stress was observed within the PDL of teeth undergoing the planned orthodontic movement, indicating effective transmission of the intended force systems to the supporting tissues. However, notable differences were identified between the two aligner designs. In the scanned aligner group, buccolingual stress within the PDL was markedly greater in the posterior teeth, particularly in the second premolars and first molars, compared with the ideal aligner group. This increased stress was attributed to expansion rebound deformation of the scanned aligners, which altered force delivery and resulted in less controlled transverse force application. Collectively these findings underscore the influence of aligner manufacturing accuracy and fit on biomechanical behavior, particularly in posterior segments, and highlight the importance of aligner design in achieving predictable and biologically favorable tooth movement [21].

Katta et al. (2023) used three-dimensional finite element analysis to evaluate the biomechanical response of a complete orthodontic system under progressively increasing forces. Simulations were performed in ANSYS Workbench by applying loads from 0.5 N to 1.0 N on bracket and tube elements, assessing displacement, strain, and stress distributions within the PDL. The patient-specific model was reconstructed from CBCT data of a 21-year-old female, with DICOM images converted into 3D anatomical structures and refined through CAD processing before incorporating orthodontic components such as brackets, tubes, and archwires. As force magnitude increased, maximum displacement, deformation, and PDL stress rose proportionally, reaching approximately 2.20 × 10^−5^ m displacement, 2.97 × 10^−3^ strain, and 9.79 × 10^7^ Pa stress at 1.0 N. These findings suggest a largely linear biomechanical response to increasing orthodontic loads. The authors highlighted that orthodontic systems exhibit elastic behavior due to both archwire flexibility and PDL compliance, reinforcing the value of FEA as a patient-specific tool for predicting force transmission and tissue response during treatment [22].

Tang et al. (2025) investigated the biomechanical effects of varying degrees of canine retraction on anterior and posterior tooth behavior using a three-dimensional finite element model with clear aligners. Different retraction scenarios were compared to assess the influences of canine movement on tooth displacement patterns and stress distribution within the dentoalveolar system. In the isolated canine retraction model, the canine exhibited distal and lingual tipping, while the incisors showed labial and mesial tipping. Conversely, in the incisor retraction model without canine movement, the incisors underwent uncontrolled lingual and distal tipping, while the canine demonstrated mesial and lingual tipping. As canine displacement increased from 0.10 mm to 0.20 mm, tooth movement patterns progressively changed: incisors exhibited reduced lingual and distal tipping with increased extrusion, while canines showed greater distal and lingual tipping accompanied by intrusion. Analysis of von Mises stress distribution in the PDL revealed distinct model-dependent patterns. Stress distribution was relatively uniform in the central incisor in the 0.25 mm group and in the lateral incisor in the 0.20 mm group. The highest stress values were observed in the lateral incisor in the 0 and 0.10 mm groups, whereas in the models with greater canine retraction, peak stresses were concentrated at the canine apex. Posterior teeth consistently demonstrated mesial inclination across all simulated conditions, with the magnitude of mesial displacement positively correlated with the extent of canine retraction. Overall, the findings indicate that the degree of canine movement plays a critical role in regulating anterior tooth inclination, vertical displacement, and stress distribution during en-masse retraction with clear aligners, underscoring the biomechanical interdependence between canine and incisor movements in orthodontic treatment planning [23].

Wang et al. (2025) investigated the biomechanical effects of torque overcorrection in clear aligners during mandibular incisor intrusion, under extraction and non-extraction conditions, using three-dimensional finite element analysis. Three clinical scenarios were simulated: premolar non-extraction with intrusion (NEI), premolar extraction with intrusion (EI), and premolar extraction with simultaneous intrusion and retraction (EIR). An intrusion of 0.25 mm was combined with torque overcorrection angles of 0° to 3°. Without overcorrection, incisors showed intrusion with lingual tipping, while canines and premolars exhibited extrusion and mesial tipping. Compressive stresses in the PDL and alveolar bone were concentrated in the lingual cervical and labial apical regions, with the EIR model presenting the highest displacement and stress levels. With increasing torque overcorrection, lingual tipping was progressively reduced, and stress distribution became more uniform. Neutralization of lingual displacement was achieved with 1° overcorrection in the NEI and EI groups, and with 3° in the EIR group. Higher overcorrection angles induced a shift toward labial tipping of incisors and distal tipping tendencies in posterior teeth, with compressive stresses relocating to the labial cervical and lingual apical regions. Overall, the study suggests that torque overcorrection can improve incisor control, reduce localized periodontal stress, and support anchorage preservation, particularly when intrusion and retraction are performed simultaneously under extraction protocols [24].

Yılmaz et al. (2025) used three-dimensional finite element analysis to evaluate the biomechanical effects of the reverse curve of Spee Ni-Ti archwires with different depths and dimensions in MBT and Roth bracket systems during mandibular leveling and alignment. Twelve static linear models were created by combining three curve depths (20, 25, and 30 mm) with two bracket systems (0.022-inch MBT and 0.018-inch Roth). Tooth displacement along the x-, y-, and z-axes, total displacement, and von Mises stress distribution in the PDL were assessed under standardized conditions. More aggressive configurations (Models 1, 2, 7, and 8), particularly those using deeper curves (30 mm) and larger wire dimensions, produced the greatest incisor displacement (up to 2.821 × 10^−4^ mm) and the highest PDL stress levels (up to 9.758 × 10^−3^ MPa). In contrast, conservative models (5, 6, 11, and 12), characterized by shallower depths (20–25 mm) and smaller wires, showed minimal displacement and lower periodontal stress. Lateral displacements were generally low, while vertical intrusion was most pronounced in Model 2 and anteroposterior movement was greatest in Models 7 and 8. Distinct biomechanical patterns were observed between bracket systems, with conservative Roth configurations demonstrating greater stability and reduced risk of PDL overload. Overall, the study highlights that bracket type, wire dimension, and curve depth significantly influence tooth movement and periodontal stress, emphasizing the need for individualized force selection to ensure biologically safe orthodontic treatment [25].

## 4. Discussion

The objective of this scoping review was to map and synthesize the existing evidence on the application of FEA in orthodontics, with particular emphasis on force distribution, tooth movement patterns, and periodontal tissue response associated with different orthodontic systems, especially clear aligners. Given the substantial heterogeneity in modeling strategies, underlying assumptions, and outcome measures across studies, a scoping review methodology was considered appropriate to comprehensively characterize the scope of current research, identify prevailing trends, and highlight gaps in the literature.

Overall, the findings support the notion that FEA is primarily used as a descriptive and exploratory tool in orthodontic research. Although FEA enables detailed visualization of stress–strain distribution and predicted tooth displacement, considerable methodological variability persists, particularly in model construction, material properties, boundary conditions, and validation approaches. This heterogeneity limits meaningful comparison between studies and constrains the direct translation of computational findings into clinical decision-making.

It should be noted that most included studies were descriptive biomechanical simulations rather than statistically driven comparative analyses. Where statistical testing was reported, methodologies were heterogeneous and not systematically comparable, reinforcing that current orthodontic FEA evidence remains primarily exploratory. Methodological heterogeneity persists largely because orthodontic FEA models differ in material assumptions, boundary conditions, and validation strategies. Common simplifications, such as linear elasticity, static loading, and omission of time-dependent remodeling, may generate discrepancies between predicted displacement patterns and real clinical outcomes, limiting direct translational applicability.

Several recurring biomechanical patterns emerged from the analyzed studies. Li et al. (2023) demonstrated that variations in the incisor–mandibular plane angle significantly influence force systems and stress distribution in mandibular incisors during clear-aligner treatment, whereas canines exhibited greater biomechanical stability. The authors also confirmed near-symmetrical behavior between homologous teeth, supporting the methodological validity of analysis a single hemi-arch. Similarly, Kang et al. (2023) reported that en-masse incisor retraction following premolar extraction produces predictable and symmetrical movement patterns, with stress concentrations predominantly localized to the cervical and apical regions of the PDL. Collectively, these findings underscore the critical influence of incisal inclination and force direction on orthodontic biomechanics [14,15].

Aligner thickness emerged as another consistent determinant of biomechanical response. Seo et al. (2021) and Jin et al. (2024) reported that thicker aligners (0.75 mm) generated slightly higher PDL stress levels than thinner aligners (0.5 mm), while differences in the magnitude of tooth displacement were minimal. This result suggests that increased aligner stiffness may enhances force transmission but at the expense of elevated periodontal stress, highlighting the need to balance mechanical efficiency with biological safety [16,17].

Anchorage control and involuntary tooth movements were also recurrent themes. Qiang et al. (2024) and Zhang et al. (2023) demonstrated that anchorage loss and tipping movements following tooth extraction are influenced by factors such as extraction patterns, step length, and torque compensation. Similarly, Zhu et al. (2024) and Tang et al. (2025) reported that distalization and canine retraction protocols frequently result in uncontrolled tipping and stress concentration in the cervical PDL, particularly when torque control is insufficient. Together, these findings emphasize the importance of precise force-system design to minimize adverse biomechanical effects during clear-aligner treatment [18,19,20,23].

The influence of force magnitude and appliance configuration was further highlighted by Katta et al. (2023) and Yılmaz et al. (2025), who demonstrated that more aggressive force systems result in greater tooth displacement but are associated with substantially increased periodontal stress. In contrast, more conservative configurations, particularly those incorporating Roth bracket systems and shallower reverse-curve depths, were linked to enhanced biomechanical stability and reduced risk of periodontal overload. Collectively, these findings emphasize the inherent trade-off between accelerating tooth movement and maintaining biological safety [22,25].

Across the included studies, a recurrent biomechanical pattern was the tendency of incisors to undergo lingual tipping during intrusion or retraction, particularly in the absence of adequate torque compensation. The incorporation of biomechanical adjuncts such as power ridges, torque overcorrection, and optimized staging protocols was shown to promote more controlled root movement and to reduce stress concentration within the PDL. These observations are consistent with existing clinical evidence indicating that effective torque control is essential to minimize adverse outcomes, including root resorption and anchorage loss. Additionally, several studies supported the methodological use of the hemi-arch model, given the near-symmetrical behavior observed in homologous teeth.

Also, clinically, controlled orthodontic extrusion has been associated with alveolar bone formation and periodontal tissue adaptation, supporting its regenerative potential in interdisciplinary treatment planning. This concept has been discussed in the context of forced eruption as a means for osseous and soft-tissue regeneration [26].

From a clinical perspective, the biomechanical patterns identified across FEA studies provide useful guidance for orthodontic treatment planning. Variables such as torque compensation, attachment configuration, aligner thickness, and staging strategies directly influence stress concentration within the periodontal ligament and the predictability of tooth movement. These findings support the role of FEA as a complementary decision-support tool to optimize force systems, minimize biomechanical risk, and enhance individualized treatment protocols, particularly in patients with periodontal risk.

### Limitations

Despite the increasing application of FEA in orthodontic research, this review identifies several important limitations. Most studies employed simplified, static, and linear modeling approaches, often without experimental or clinical validation. Key biological processes, such as bone remodeling, periodontal adaptation, and time-dependent tissue responses, remain insufficiently represented. Furthermore, the absence of standardized protocols for model construction, material property assignment, and outcome reporting limits comparability across studies and reduces the clinical applicability of findings.

Although FEA enables detailed evaluation of the distribution internal stresses and predicted tooth movement, the majority of included studies were based on isolated virtual models lacking longitudinal clinical validation. Although finite element analysis provides detailed visualization of stress distribution and displacement patterns, the clinical applicability of these findings remains limited. Moreover, complex biological phenomena, including bone remodeling, inflammatory response, and individual variability in tissue properties, cannot be fully replicated in computational simulations. Therefore, FEA outcomes should be interpreted primarily as theoretical biomechanical estimations rather than direct clinical predictors, highlighting the need for longitudinal clinical validation and biologically adaptive modeling approaches.

Also, a further limitation is that relatively few studies incorporated experimental or clinical validation alongside computational simulations. Hybrid in silico and in vivo approaches represent a critical future direction to strengthen confidence in FEA-derived predictions and enhance clinical applicability.

In addition to limitations inherent to finite element simulations, this scoping review is subject to methodological constraints typical of evidence mapping studies, including the inability to formally assess study quality or risk of bias. Furthermore, most FEA models assume simplified material properties, static force application, limited patient variability, and absence of long-term biological adaptation, which restricts extrapolation to real-world orthodontic scenarios.

## 5. Conclusions

This scoping review mapped the existing evidence on the application of FEA in orthodontic biomechanics, confirming its widespread use as a supportive research methodology. The findings indicate that appliance design, aligner thickness, attachment configuration, applied torque, and periodontal conditions are key determinants of tooth movement efficiency and biological safety. However, FEA is currently employed predominantly for descriptive purposes, focusing on visualization of stress and strain distribution rather than predictive clinical outcomes. Future research should prioritize methodological standardization, rigorous experimental and clinical validation, and the development of biologically realistic, time-dependent models to enhance the translational relevance of FEA in contemporary orthodontic practice.

## Figures and Tables

**Table 1:** Main characteristics from included studies.

Author/Year	Type of Study	Orthodontic Appliance	FEA Model/Software	Type and Magnitude of Force	Observed Movement	Conclusion
Li et al., 2023 [14]	3D simulation	Aligners (intrusion of lower incisors)	3D/HyperMesh (Altair Engineering Inc., Troy, MI, USA)	0.2 mm intrusion/IMPA 90–110°	Compression of the lingual fossa and apex; labial inclination of roots; extrusion of canines	The greater the IMPA, the more the direction of force is reversed; roots always tend to tilt labially
Kang et al., 2023 [15]	3D simulation	Aligners + premolar extraction	3D/Not specified	200 g intrusion/60–90° angles	Lingual tilt and extrusion of incisors; intrusion of canines; mesialization of posterior teeth	More vertical forces achieve greater intrusion; risk of root resorption in incisors
Seo et al., 2021 [16]	3D simulation	Aligners with different thicknesses	3D/ABAQUS (v6.14, Dassault Systèmes Corp)	Induced pre-stress/0.75 mm vs. 0.05 mm	Lingual tilt and axial rotation; displacement of the center of rotation	Aligner thickness influences PDL stress and center of rotation, but both allow sufficient movement
Jin et al., 2024 [17]	3D simulation + experimental model	Regular aligners vs. reinforced frame aligners	3D/HyperMesh and ABAQUS (Dassault SIMULIA, Providence, RI, USA)	Anterior retraction/[Unspecified]	Less mesial tipping in premolars and molars; greater retraction in canines	Reinforced structure improves force distribution, reduces anchor loss and improves retraction
Qiang et al., 2024 [18]	3D simulation	Aligners + extraction of 1st or 2nd premolars	3D/ANSYS (Altair, Troy, MI, USA)	Mass retraction/[Unspecified]	Greater lingual inclination in anterior teeth; milder mesial tipping in model with first premolar extraction	Extraction of 1st premolars generates better stress distribution and less loss of posterior anchorage
Zhang Hui et al., 2023 [19]	3D simulation	Aligners + maxillary expansion	3D/ANSYS	Expansion/stride 0.1–0.3 mm + torque 0–2°	Buccal inclination of posterior teeth; lingual inclination and extrusion of anterior teeth	Torque compensation improves body movement; it should be adjusted according to the patient and clinical objective
Zhu et al., 2024 [20]	3D simulation	Aligners + 5 molar movement configurations	3D/CBCT-based model/ANSYS	Distalization 0.25 mm + extrusion/expansion 0.15 mm	Lip tipping of incisors; less distalization in E configuration; increased pressure on anterior PDL	Configuration E generates greater stress on incisors; periodontal status should be considered before applying clinical protocols
Mao et al., 2023 [21]	3D simulation + statistical analysis	Scanned vs. ideal aligners	3D/CBCT + digital models (2016, SIMULIA Co, USA)/ABAQUS	ERD by thermoforming/[Not specified]	Increased coronal displacement in lateral incisors, canines, premolars, and molars; stress on posterior PDL	Expansion deformation of thermoformed aligners can cause unwanted movement and affect clinical outcomes
Katta et al., 2023 [22]	3D simulation based on real patient	Brackets + tubes + orthodontic wires	3D/InVesalius (CTI, Campinas, Brazil) + Geomagic (Morrisville, NC, USA) + ANSYS Workbench (Ansys Inc., Canonsburg, PA, USA)	0.5–1 N/multiple loads	Displacement, deformation, and stress diagrams; elastic behavior of the orthodontic system	The orthodontic system is elastic due to the wires and periodontal ligament; FEM allows for personalized analysis
Tang et al., 2025 [23]	3D simulation	Aligners + mass retraction	3D/ANSYS^® ANSYS®, Pennsylvania, PA, USA^	Incisor retraction 0.15 mm + intrusion 0.10 mm; canine retraction 0–0.30 mm	Lingual tipping of incisors without canine retraction; mesial tipping of posterior teeth correlated with canine retraction	Increasing canine retraction reduces lingual tipping of incisors; the designed movements affect adjacent teeth
Wang et al., 2025 [24]	3D simulation	Aligners + torque overcorrection	3D/ANSYS (17.0 software (ANSYS, Pennsylvania, PA, USA))	Intrusion 0.25 mm + torque 0–3°	Tongue tipping without correction; lip tipping and improved stress distribution with compensated torque	Overcorrection torque reduces tongue tipping and improves stress distribution in the PDL and alveolar bone during intrusion and retraction
Yilmaz et al., 2025 [25]	3D simulation	Reverse-curved archwires with Roth and MBT brackets	3D/[Not Specified]	Static forces/depth 20–30 mm	Greater displacement and stress in PDL with deeper and thicker arches; more conservative forces with less depth	Arch selection should consider periodontal health; aggressive forces may compromise treatment safety

## Data Availability

No new data were created in this study. Data sharing is not applicable for this study.
